# Comorbidities and psychosocial factors as correlates of self-reported falls in a nationwide sample of community-dwelling people aging with HIV in Germany

**DOI:** 10.1186/s12889-021-11582-2

**Published:** 2021-08-12

**Authors:** Jochen Drewes, Jennifer Ebert, Phil C. Langer, Dieter Kleiber, Burkhard Gusy

**Affiliations:** 1 Public Health: Prevention and Psychosocial Health Research, Freie Universität Berlin, Habelschwerdter Allee 45, 14195 Berlin, Germany; 2 International Psychoanalytic University, Stromstr. 3b, 10555 Berlin, Germany

**Keywords:** Falls, Aging, Geriatric syndromes, HIV, HIV stigma, Social support, Socioeconomic status, Psychosocial factors

## Abstract

**Background:**

Falls are a frequent health problem with potentially severe consequences among the elderly. Due to the aging HIV population, there is a growing interest in falls as a geriatric syndrome in HIV research and clinical practice. Previous studies found rather high prevalences of falls in this population and focused on biomedical and demographic risk factors for falls. Psychosocial risk factors like stigma, social support or loneliness were not previously assessed as correlates of fall events in this population.

**Methods:**

We assessed self-reported fall frequency in the past 12 months in a nationwide sample of 897 community-dwelling people aged 50 years or older living with HIV in Germany using a cross-sectional study design. We calculated odds of any fall for sociodemographic and HIV-related variables in bivariate analyses and for comorbidities, and psychosocial variables in bivariate and adjusted analyses.

**Results:**

Eighteen percent of our participants reported at least one fall in the preceding 12 months, 12 % reported recurring falls. A lower socioeconomic status, being single and living alone were significantly associated with a higher risk for falling. An AIDS diagnosis was related to fall risk, but time since diagnosis and a detectable viral load were not. Reporting at least one comorbidity increased fall risk in our sample 2.5 times (95% CI: 1.59; 3.97). The strongest association with fall risk was found for diseases of the central nervous system, heart disease, rheumatism, osteoporosis, and chronic pain. Experienced HIV stigma (AOR: 2.11; 95% CI: 1.58; 2.83) and internalized HIV stigma (AOR: 1.43; 95% CI: 1.12; 1.85), as well as social support (AOR: .92; 95% CI: .86; .99) and loneliness (AOR: 1.51; 95% CI: 1.22; 1.87) were significantly related to fall risk in bivariate and adjusted analyses.

**Conclusions:**

We found a low prevalence of falls in our sample of community-dwelling people aging with HIV. Our results show evidence for a strong association between comorbidity and falling, and between psychosocial factors and falling. Especially the strong association between experienced HIV stigma and fall risk is noteworthy and adds falls to the list of health outcomes affected by HIV stigma.

## Background

Falls are a frequent health problem with potentially severe consequences among the elderly. Nationally representative studies in Germany reported annually fall rates between 26 and 39% for women and between 16 and 30% for men aged 65 years and older [1]. Falls can result in severe injury leading to disability, hospitalization, loss of independence, and an elevated mortality risk [2]. In geriatrics, falls are understood as a geriatric syndrome - a class of conditions that have in common to be highly prevalent among the elderly, to have multifactorial causes, and to result in substantial morbidity and poor health outcomes [3].

### Falls in people aging with HIV

While the population of people living with HIV/AIDS is aging, there is a growing interest in geriatric syndromes in HIV research and clinical practice. Authors have argued that risk factors for geriatric syndromes are widespread, and geriatric syndromes are common among people aging with HIV/AIDS (PAWH) [4]; and that PAWH are affected by these syndromes at a younger age compared to the general population [5]. Greene and colleagues, who assessed eleven geriatric syndromes among PAWH, reported high prevalences with 54% of their subjects being diagnosed with at least two geriatric syndromes. Falls were widespread in this sample with 26% of the participants reporting at least one fall in the recent 12 months [4]. For the same time frame but different age groups, sample compositions and sample sizes diverging fall rates were reported ranging between 18% [6] and 41% [7]. A 41% fall rate was also found in two longitudinal studies which followed their participants for 2 years [8], with one reporting a rate of 10% of these falls being complicated by “an injury leading to medical evaluation” [9]. Studies comparing fall rates between people living with HIV (PLWH) and uninfected individuals did not show statistically significant differences between both groups [10], while fall rates were actually higher in PLWH in one study [11]. Similarly, studies showed that neither the HIV status itself nor HIV disease- or treatment-related characteristics were significantly associated with fall events [8, 12]. Instead, risk factors for falls in this population were largely the same as the risk factors that were identified for non-infected individuals [13]. Falls in PLWH can result in serious consequences, fall-related fractures showed a prevalence of 3.8 to 8% among PLWH fallers [10], in one study every fifth faller sought medical attention because of a fall [6]. A recent study reported a strong association between falls and mortality in middle-aged adults with HIV [14].

### Psychosocial risk factors for falls among the elderly

Apart from sociodemographic variables, no research was yet published on the relationship between psychosocial risk factors and fall risk among PLWH. Research on psychosocial risk factors in this area is in general rare [15] and research results are far from being conclusive. Social support/social integration is the most frequently studied psychosocial risk factor, with studies showing positive associations [16, 17], negative associations [17], and null findings [18] between different operationalizations of social support and fall risk. Whereas loneliness was consistently associated with fall risk in previous studies [18, 19].

### Present study

The purpose of the present study is to describe fall prevalence in a nationwide convenience sample of community-dwelling PAWH living in Germany and to evaluate factors associated with fall risk in this population. We will focus on the analysis of comorbid conditions, sociodemographic and psychosocial factors as correlates of fall risk in this population.

## Methods

### Study design

The cross-sectional, exploratory study ‘50plushiv: Psychosocial aspects of aging with HIV/AIDS in Germany’ was conducted in 2013–14 to assess health status, health behaviors, and living conditions of people aging with HIV/AIDS in Germany with a particular focus on psychosocial variables. While we used quantitative and qualitative methods in this study, this paper only examines the quantitative data from a large online and paper pencil questionnaire. Further information on the methods were published earlier [20]. The information we obtained from the participants is exclusively self-reported. All participants provided informed consent and the study was approved by the Freie Universität Berlin ethics committee.

### Participants

Eligible were all individuals diagnosed with HIV who were 50 years and older and resided in Germany. Of 907 participants who completed the questionnaire and met the eligibility criteria we eliminated 10 participants due to missing data in the falls variable resulting in a study sample of *n* = 897.

### Variables

We used sociodemographic variables like age, sex, sexual orientation, relationship and living status, and HIV-related variables like duration of HIV infection, HIV RNA and whether the participant was ever diagnosed with AIDS along with the following variables.

#### Falls

Participants were asked if and how often they fell, stumbled, or slipped without any evident reason in a way they lost balance and landed on the ground or a lower level in the previous 12 months. We calculated prevalences for any fall and recurring falls (> 1 fall), with any falls being our primary outcome which we used for the calculations of the odds ratios.

#### Comorbidity

We assessed the prevalence of 20 physical comorbid diseases and depression the participant is currently treated for (see [20] for further information on operationalization and prevalences; note that because of low prevalences comorbid cancer diseases were not included in these analyses). A comorbidity sum index was built by adding all 21 conditions the participant is currently treated for.

#### Socioeconomic status

We assessed SES using a multi-dimensional index developed by Germany’s national public health institute [21] that included the three dimensions of SES education, occupation, and income. Scores for all dimensions were combined into a single SES score with a range from 1 to 7, with 1 indicating the lowest possible SES.

#### Experienced HIV stigma

To assess experienced HIV stigma we used a self-constructed scale. Our scale measured the frequency with which different forms of HIV-related stigmatization and discrimination were experienced by the respondent. Scores had a possible range from 0 to 4 with higher scores indicating more experienced HIV stigma. Internal consistency for this 6-item scale was good: Cronbach’s α = .80. For more information on this scale and the original items see [22].

#### Internalized HIV stigma

We assessed internalized HIV stigma with an adapted version of the negative self-image subscale of the HIV Stigma Scale [23, 24]. See [22] for more information of the adaptation process. Possible scores of our internalized HIV stigma scale range between 1 and 4 (expressing the highest possible extent of internalized HIV stigma). The 6-item scale showed a good internal consistency: Cronbach’s α = .89.

#### Adversarial growth

Adversarial growth was measured using an abbreviated version of the Silver Lining Questionnaire (SLQ) [25, 26]. We selected eight items from the original 38-item instrument and used a 4-point Likert scale, with higher scores indicating more adversarial growth. The procedure is described in detail elsewhere [22]. Our 8-item scale showed excellent internal consistency with Cronbach’s alpha = .90.

#### Social support

To assess social support we used the OSLO 3 Social Support Scale (OSSS-3) [27]. The OSSS-3 is a sum index measuring the perceived availability of social support. The OSSS-3 score is calculated as the sum of the raw scores of the three items, with a possible range between 3 and 14, with higher scores indicating more social support. We changed the wording of the last item from “neighbors” to “friends”. Internal consistency of our 3-item scale was acceptable with Cronbach’s α = .76.

#### Loneliness

We used an abbreviated version of the UCLA Loneliness Scale [28] to assess loneliness. We selected only the three highest loading items on the factor ‘feelings of loneliness’ as part of a three factor solution proposed by Döring and Bortz [29]. Scores ranged between 1 and 4 with higher scores indicating more feelings of loneliness. Our 3-item scale showed good internal consistency with Cronbach’s α = .86.

### Statistical methods

All statistical analyses were conducted using the SPSS24 software. Unadjusted odds ratios for dichotomous predictors were calculated using the option that is provided in the crosstab function in SPSS. Unadjusted odds ratios for metric predictors and adjusted odds ratios for all predictors were conducted using binary logistic regression analyses. For calculations of all odds ratios, we compared participants with at least one fall with participants who did not fall. To calculate adjusted odds ratios for comorbidities we used three variables as covariates in the binary logistic regression analyses which were either related to falls or comorbidity burden in our sample and that we assumed to potentially confound the association between fall risk and comorbidity (age, duration of HIV infection, and an AIDS diagnosis). For the adjusted logistic regression analyses of psychosocial variables, we further used the comorbidity sum index as a covariate. We chose a significance level of *p* < .05 for all analyses.

## Results

We used the data of 897 subjects of whom 87% were male. The mean age was 57 years (SD = 6.7), with the majority of 71% being between 50 and 59 years old. Subjects lived on average 17 years with HIV (time since diagnosis; SD = 8.6), and 93% reported an undetectable HIV1 RNA viral load (see Table 1).

**Table 1:** Sample characteristics (*n* = 897)

	N (%)	Mean (Standard deviation)	Range
Falls			
none	732 (81.6%)		
single fall	43 (4.8%)		
recurring falls	109 (12.2%)		
falls without frequency	13 (1.4%)		
Age		57.3 (6.7)	50–83
50–59 years	638 (71.1%)		
60–69 years	191 (21.3%)		
70 years and older	68 (7.6%)		
Sex			
male	781 (87.1%)		
female	116 (12.9%)		
Sexual orientation			
heterosexual men	76 (8.5%)		
homo−/bisexual men	699 (78.5%)		
heterosexual women	107 (12.0%)		
homo−/bisexual women	8 (0.9%)		
Education			
10 years or less	484 (55.3%)		
more than 10 years	392 (44.7%)		
Socioeconomic status		12.4 (4.0)	3.3–21
Duration of HIV-infection		16.8 (8.6)	1–32
1–5 years	104 (11.6%)		
6–10 years	150 (16.8%)		
11–20 years	310 (34.6%)		
21 years and longer	331 (37.0%)		
HIV-1 RNA			
detectable	61 (7.1%)		
undetectable	799 (92.9%)		
AIDS diagnosis			
yes	296 (34.8%)		
no	554 (65.2%)		
Social Support		10.0 (2.5)	3–14
Loneliness		3.0 (.9)	1–4
Experienced HIV stigma		0.5 (.6)	0–4
Internalized HIV stigma		1.6 (.7)	1–4
Adversarial growth		2.3 (.8)	1–4

In total 165 subjects (18%) reported at least one fall in the preceding 12 months, with 28% of the fallers reporting just one fall (43 subjects) and 72% reporting recurring falls (109 subjects, in total 12% of our sample; 13 participants reported falling in the preceding 12 months but did not specify the amount of falls they experienced). People with a higher SES had a lower risk of falling. Participants living alone and participants not in a relationship had higher odds of falling, as well as participants who were diagnosed with AIDS. Age, being female, and a homosexual identity were not significantly associated with falling, as well as time since HIV diagnosis and a detectable HIV-1 RNA viral load (see Table 2).

**Table 2:** Odds of any fall in the last 12 months for sociodemographic and HIV-related characteristics (*n* = 897)

	No Falls (*n* = 732)	Any Falls (*n* = 165)	Odds Ratio
Age^a^ (years)	57.3 (6.7)	57.6 (6.7)	1.01 (.98; 1.03)
Sex: female	88 (12%)	28 (17%)	1.50 (.94; 2.38)
Sexual orientation: homosexual	578 (80%)	129 (78%)	.91 (.60; 1.38)
Socioeconomic status^a^	12.6 (4.1)	11.5 (3.8)	**.93 (.89; .98)**
Relationship status: not partnered	321 (44%)	90 (55%)	**1.55 (1.10; 2.17)**
Living status: alone	381 (52%)	103 (64%)	**1.60 (1.13; 2.27)**
Duration of HIV infection^a^ (years)	16.5 (8.4)	17.9 (9.1)	1.02 (.99; 1.04)
HIV-1 RNA: detectable	68 (10%)	21 (14%)	1.48 (.88; 2.50)
AIDS diagnosis: yes	225 (32%)	71 (46%)	**1.79 (1.26; 2.55)**

Note: *ART* antiretroviral therapy, *RNA* ribonucleic acid. Odds Ratios in bold are significant at the .05 level

^a^ For metric variables table shows arithmetic mean and standard deviation

### Comorbidity and fall risk

Comorbidity was associated with risk for falling in our sample. Reporting at least one comorbidity was associated with a 2.5-fold risk for falling compared to reporting no comorbidity in adjusted logistic regression analyses (95% CI: 1.59; 3.97). The number of reported comorbidities was also significantly associated to falling in adjusted analysis: with each added condition, the odds of falling increased 1.2 times (95% CI: 1.13; 1.30). Table 3 shows unadjusted and adjusted odds ratios for all comorbid conditions.

**Table 3:** Odds of any fall in the last 12 months for self-reported comorbid conditions the patient is currently treated for (*n* = 897)

	No Falls	Any Fall	Odds Ratio	Adjusted Odds Ratio
Diseases of the central nervous system (e.g., epilepsy, Parkinson disease)	20 (2.8%)	20 (12.7%)	**5.08 (2.66; 9.69)**	**4.93 (2.52; 9.64)**
Heart disease (e.g., heart failure, heart arrhythmia, valvular heart disease)	47 (6.5%)	31 (19.1%)	**3.41 (2.09; 5.58)**	**3.13 (1.85; 5.28)**
Rheumatism	34 (4.7%)	22 (13.8%)	**3.24 (1.84; 5.71)**	**3.00 (1.165; 5.45)**
Osteoporosis	26 (3.6%)	16 (9.9%)	**2.95 (1.54; 5.63)**	**2.84 (1.43; 5.63)**
Chronic pain	106 (14.7%)	51 (31.3%)	**2.64 (1.79; 3.90)**	**2.82 (1.87; 4.24)**
Lipodystrophy, lipoatrophy	22 (3.1%)	16 (9.8%)	**3.45 (1.77; 6.73)**	**2.46 (1.18; 5.13)**
Severe problems with attention/memory	30 (4.1%)	17 (10.4%)	**2.69 (1.45; 5.01)**	**2.40 (1.25; 4.60)**
Polyneuropathy	87 (12.0%)	46 (27.9%)	**2.84 (1.89; 4.27)**	**2.37 (1.54; 3.67)**
Myocardial infarction	32 (4.4%)	18 (11.2%)	**2.72 (1.48; 4.98)**	**2.32 (1.22; 4.39)**
Kidney disease (e.g., kidney failure)	27 (3.7%)	11 (6.8%)	1.89 (.35; 1.91)	**2.21 (1.03; 4.73)**
Lung disease (e.g., asthma, COPD)	77 (10.7%)	32 (19.8%)	**2.07 (1.31; 3.25)**	**2.12 (1.33; 3.38)**
Diabetes mellitus	47 (6.5%)	22 (13.8%)	**2.29 (1.34; 3.93)**	**1.91 (1.07; 3.41)**
Disease of the gastrointestinal system (e.g., peptic ulcer disease, inflammatory bowel disease, gastritis)	81 (11.1%)	32 (19.9%)	**1.98 (1.26; 3.11)**	**1.88 (1.17; 3.00)**
Stroke	21 (2.9%)	10 (6.2%)	**2.21 (1.02; 4.78)**	1.77 (.78; 4.00)
Coronary artery disease	56 (7.7%)	23 (14.3%)	**1.99 (1.18; 3.34)**	**1.76 (1.01; 3.06)**
Peripheral artery occlusive disease	31 (4.3%)	12 (7.5%)	1.81 (.91; 3.60)	1.67 (.81; 3.41)
Depression	136 (18.7%)	47 (28.5%)	**1.73 (1.18; 2.55)**	**1.62 (1.07; 2.46)**
Hypertension	239 (32.8%)	68 (42.5%)	**1.51 (1.07; 2.15)**	1.43 (.98; 2.08)
Liver disease (e.g., liver cirrhosis)	50 (6.9%)	11 (6.7%)	.97 (.49; 1.91)	.97 (..49; 1.94)
Hepatitis c	21 (2.9%)	5 (3.1%)	1.07 (.40; 2.88)	.81 (.42; 3.11)
Hepatitis b	34 (4.8%)	1 (0.6%)	**.12 (.02; 0.91)**	.**12 (.02; .90)**
Any comorbidity (vs. none)	472 (64.5%)	138 (83.6%)	**2.81 (1.81; 4.37)**	**2.51 (1.59; 3.97)**
Comorbidity sum index^a^	1.9 (2.2)	3.4 (3.1)	**1.24 (1.16; 1.32)**	**1.22 (1.13; 1.30)**

Note: Adjusted for age, duration of HIV infection, and AIDS diagnosis. Coefficients in bold are significant at the .05 level

^a^Table shows arithmetic mean and standard deviation

### Psychosocial factors and fall risk

All psychosocial factors, except adversarial growth, were significantly associated with fall risk in bivariate and adjusted logistic regression analyses, as shown in Table 4. More social support was associated with reduced odds of falling (AOR: .92; 95% CI: .86; .99), whereas more feelings of loneliness were related to an increased odds for falling (AOR: 1.51; 95% CI: 1.22; 1.87). More experienced HIV stigma had the strongest relationship with fall risk among the psychosocial variables (AOR: 2.11; 95% CI: 1.58; 2.83). Higher levels of internalized HIV stigma (OR: 1.43; 95% CI: 1.12; 1.85) were also associated with higher odds of falling.

**Table 4:** Odds of any fall in the last 12 months for psychosocial factors (*n* = 897)

	No Falls	Any Fall	Odds Ratio	Adjusted Odds Ratio^a^
Social Support	10.2 (2.4)	9.4 (2.8)	**.89 (.83; .95)**	**.92 (.86; .99)**
Loneliness	1.9 (.8)	2.4 (.9)	**1.68 (1.38; 2.04)**	**1.51 (1.22; 1.87)**
Experienced HIV stigma	.4 (.5)	.8 (.7)	**2.25 (1.73; 2.93)**	**2.11 (1.58; 2.83)**
Internalized HIV stigma	1.5 (.7)	1.7 (.8)	**1.46 (1.17; 1.83)**	**1.43 (1.12; 1.85)**
Adversarial Growth	2.3 (.7)	2.3 (.8)	.98 (.79; 1.23)	–

Note: Coefficients in bold are significant at the .05 level

^a^Adjusted for age, duration of HIV infection, AIDS diagnosis, and sum of comorbidities

## Discussion

In our sample of community-dwelling PAWH between 50 and 83 years, almost every fifth participant reported at least one fall in the preceding 12 months and 12% reported recurring falls. Our fall prevalence of 18% is on the lower end of the range of fall rates reported by other studies in this population, with studies reporting fall rates as high as 41% (see introduction). Considerable differences in fall rates between studies are not unusual and are seen also in samples of non-HIV-infected individuals [1].

Only four of the sociodemographic and HIV-related factors were significantly associated with falls, socioeconomic status, relationship and living status, and a self-reported AIDS diagnosis. Variables like age and sex, which proved to be associated with falls in several studies, were not associated with falls in our sample, maybe due to the specific composition of our sample. Also a detectable viral load was not associated with fall risk in our sample, a result that is consonant with previous results, except for a recent study that found associations with HIV-RNA and fall risk [9]. Comorbidity increased the risk for falls significantly, and 15 of the 20 physical comorbid conditions plus depression had significantly increased odds of falling. Reporting at least one comorbidity was associated with a 2.5 higher odd for falling. And our comorbidity sum index showed that each additional comorbidity was associated with a 1.2 fold increase in fall risk. Comorbidity was in other studies with aging HIV populations also associated with an increased fall risk (see 10) and with an increased risk for frailty [4, 30] another geriatric syndrome. Comorbidity further increases potential polypharmacy in PLWH [31], which was found to be a strong predictor of falls in this population [32].

The most novel findings from our study concern the associations between psychosocial variables and fall risk in PAWH. We found significant associations between fall risk and experienced HIV stigma, internalized HIV stigma, social support, and feelings of loneliness, but no evidence for an association with adversarial growth in our sample. Social support and loneliness were related to falls in general population samples, with social support showing more inconsistent results than loneliness (see introduction). Higher levels of feelings of loneliness increased fall risk in our sample, while higher levels of social support decreased fall risk. The adverse effects of loneliness and a lack of social support on health outcomes are widely documented [33, 34]. Similar pathways how both variables influence physical health outcomes were suggested: via behavioral mechanisms like health behaviors and adherence to medical regimens, via psychological processes like depression, feelings of control and appraisal processes and coping mechanisms, and via biological processes like cardiovascular, endocrine and immune function [35, 36]. Several of these mechanisms offer a possible explanation for the link between social support and loneliness and falls we found in our sample. For example, social support is known to be protective of depression [37], whereas loneliness is a predisposing factor for depression [38], depression was in our sample associated with a 1.6 increase in fall risk and predictive of falls in other studies too [39]. Similarly, social support and loneliness have been linked to cardiovascular functioning [35, 36], and (cardio)vascular function was suggested to play a prominent role in the etiology of geriatric syndromes including falls [40].

The highest odds for falling among the psychosocial variables was found for experienced HIV stigma, while internalized HIV stigma had a still significant but smaller association. The relationship between fall risk and HIV stigma or any other health-related stigma was to our knowledge not yet studied. In general, the adverse effects of HIV stigma on physical and other health outcomes are well established [41, 42]. Similarly to social support and loneliness, stigma does not directly influence physical health outcomes, the effect is hypothesized to be mediated by psychological, social, behavioral and stress processes [43, 44]. Possible pathways how HIV-related stigma influences falling include maladaptive coping strategies, like alcohol and drug use, chronic stress that is associated with adverse physiological responses and depression, and social isolation which is associated with low social support and depression (see [43]), with each mechanism being linked to fall risk. The stronger relationship between experienced HIV stigma and fall risk compared to the relationship between internalized HIV stigma and fall risk can be seen as further evidence for the hypothesis that experienced HIV stigma is stronger related to physical health outcomes than internalized HIV stigma [22, 45].

### Limitations

Our study has strengths and limitations. The main strength of our study is the relatively big sample of PAWH we were able to realize, and the extensive information regarding sociodemographic variables, medical diagnoses, and psychosocial variables we obtained from our participants. Unfortunately, we had to rely on self-administered questionnaires with all obtained information being self-reported by the participants. We assessed fall history also by self-report and retrospectively. While prospective methods of data collection are recommended [46], a study comparing retrospectively and prospectively collected data was not able to find differences in fall incidence between both methods [1]. Further, we were not able to realize a probability sample of PAWH in Germany. The fall prevalence in our sample can thus not be generalised to the population of PAWH in Germany. It is possible that the self-selected sampling led to biases in our sample regarding the health condition of participants, with the risk of underestimating the fall prevalence in the population.

The cross-sectional design of our study does not allow us to infer causality between predictors and our outcome variable, and our results should be interpreted with caution until they are replicated by studies with a longitudinal design. Indeed, some of our psychosocial factors are conceptualized in other studies as possible consequences of falls. For example, in a large longitudinal study falls subsequently decreased social participation and increased social support [47]. Also, the association between stigma and falls is not necessarily unidirectional. In a recent qualitative study among PLWH with a history of falls, a group of participants reported trying to hide their falls because falls were viewed as markers of ill-health, and a potential sign revealing their HIV status to others, creating more possibilities to experience HIV-related stigma. In addition, falls on their own seem to be viewed as stigmatizing events by the elderly regardless of HIV status [48].

Our socio-epidemiological study was not especially designed to assess risk factors for falls and thus lacks several variables that were identified in previous studies as important risk factors for falls, like problems with gait or balance, mobility, or specific medications. Some of these variables may have been important to add to our logistic regression model as covariates, other variables may be mediating the relationship between psychosocial factors and fall risk. Due to the cross-sectional design of our study, we did not test for potential mediation processes.

## Conclusions

Our study is the first to analyze fall risk among PAWH in Germany and fall prevalence in our large sample of community-dwelling PAWH was on the lower end of rates found in similar populations. We found strong evidence for an association between physical and psychiatric comorbid conditions and fall risk, with 15 of 21 comorbidities being related to an increased fall risk in our sample. We were the first to systematically study psychosocial factors as risk factors of fall events in the elderly. While our results need confirmation from future studies employing longitudinal study designs and assessing a wider range of risk factors of falls, we found experienced HIV stigma, internalized HIV stigma, social support, and loneliness to be related to fall risk in our sample. With experienced HIV stigma showing the strongest association with fall risk, our study adds falls to the long list of health outcomes being affected by HIV-related stigma.

## Data Availability

The dataset generated and analyzed during the current study is available from the corresponding author on reasonable request.

## Abbreviations

HIV: Human immunodeficiency virus
AIDS: Acquired immune deficiency syndrome
PAWH: People aging with HIV/AIDS
PLWH: People living with HIV/AIDS
RNA: Ribonucleic acid
SES: Socioeconomic status
SLQ: Silver Lining Questionnaire
OSSS-3: OSLO Social Support Scale
UCLA: University of California, Los Angeles
SD: Standard deviation
CI: Confidence interval
OR: Odds ratio
AOR: Adjusted odds ratio
